# Fish species composition, distribution and community structure in the lower reaches of Ganjiang River, Jiangxi, China

**DOI:** 10.1038/s41598-019-46600-2

**Published:** 2019-07-12

**Authors:** Maolin Hu, Chaoyang Wang, Yizhen Liu, Xiangyu Zhang, Shaoqing Jian

**Affiliations:** 1 School of Life Sciences, Nanchang University, Nanchang, Jiangxi 330031 P. R. China; 2 Ministry of Education Key Laboratory of Poyang Lake Environment and Resource Utilization, Nanchang University, Nanchang, Jiangxi 330031 P. R. China; 3 Jiangxi Key Laboratory of Aquatic Animal Resource and Utilization, Nanchang University, Nanchang, Jiangxi 330031 P. R. China

**Keywords:** Ichthyology, Biodiversity

## Abstract

The Ganjiang River (length: 823 km; drainage area: 82,809 km^2^) is the largest river that flows into Poyang Lake and an important tributary of the Yangtze River. In this study, fish fauna were collected from 10 stations in the lower reaches of the river (YC: Yichun, XY: Xinyu, SG: Shanggao, GA: Ganan, ZS: Zhangshu, FC: Fengcheng, NC: Nanchang, QS: Qiaoshe, NX: Nanxin, CC: Chucha) from March 2017 to February 2018. The species composition and distribution as well as spatio-temporal variation in biodiversity and abundance were then examined. Overall, 12,680 samples comprising15 families and 84 species were collected, the majority of which belonged to the Order Cypriniformes (69.05% of the total species collected) and Cyprinidae (64.29%). Moreover, of these 84 species, 36 (42.86%) were endemic to China. Dominant species were *Cyprinus carpio* (index of relative importance (*IRI*): 17.19%), *Pseudobrama simoni* (*IRI*: 10.81%) and *Xenocypris argentea* (*IRI*: 10.20%). Subsequent cluster analysis divided the samples into three significantly different groups by sample site. Meanwhile, Margalef species richness and Shannon−Wiener diversity indices were both low, and along with analyses of abundance-biomass curves suggested moderate disturbance. Current threats to the conservation of fish biodiversity in the lower reaches were also reviewed and management solutions suggested. The results will help form the basis for reasonable exploitation and protection of freshwater fish in the lower reaches of the Ganjiang River.

## Introduction

China is the most populous country in the world and the fourth largest by area, as well as one of 17 megadiversity countries^1^. Approximately 1323 species of freshwater fish fauna are found in China, representing 9% of the global total^2^, and of these, 877 are endemic^2^.

The Yangtze River is the largest river in China and third longest in the world. With more than 3000 tributaries and 4000 lakes^3^, the Yangtze River forms a complete riverine-lacustrine network. So far, 426 species and subspecies have been recorded in the river basin, 322 of which are thought to be endemic^2,4^. The Yangtze River forms a complex river-lake ecosystem with Poyang Lake, the largest freshwater lake in China, its five tributaries (Ganjiang River, Xinjiang River, Fuhe River, Raohe River and Xiuhe River). Poyang Lake is situated in Jiangxi Province, which harbours 220 freshwater fish species, of which 131 are endemic to China^5^. Of the rivers flowing into the lake, the Ganjiang River is the largest. It is also one of the most important tributaries (7th largest) flowing into the Yangtze River. To date, 118 fish species have been recorded in this river alone^6^ with 12 spawning grounds of four domestic fish species as well as one breeding area of *Macrura reevesii* also having been reported^5,7^. The Ganjiang River therefore plays a significant role in maintaining and replenishing fish resources in both Poyang Lake and the Yangtze River.

Fish are the most diverse of all vertebrate groups; however, they are also the most threatened after amphibians^8–10^. Freshwater biodiversity, in particular, is currently facing a global crisis, with many of the world’s most species-rich, threatened and valuable fish lineages afforded no legal protection^11^. Various fishery, hydropower and navigation activities currently exploit China’s extensive freshwater resources, with detrimental effects on biodiversity. Meanwhile, although the threats from hydroelectric use, pollution and habitat degradation have been extensively documented^12,13^, little remediation has been carried out and as a result, many endemic and iconic fish species are now either endangered or at high risk of extinction.

**Figure 1:**
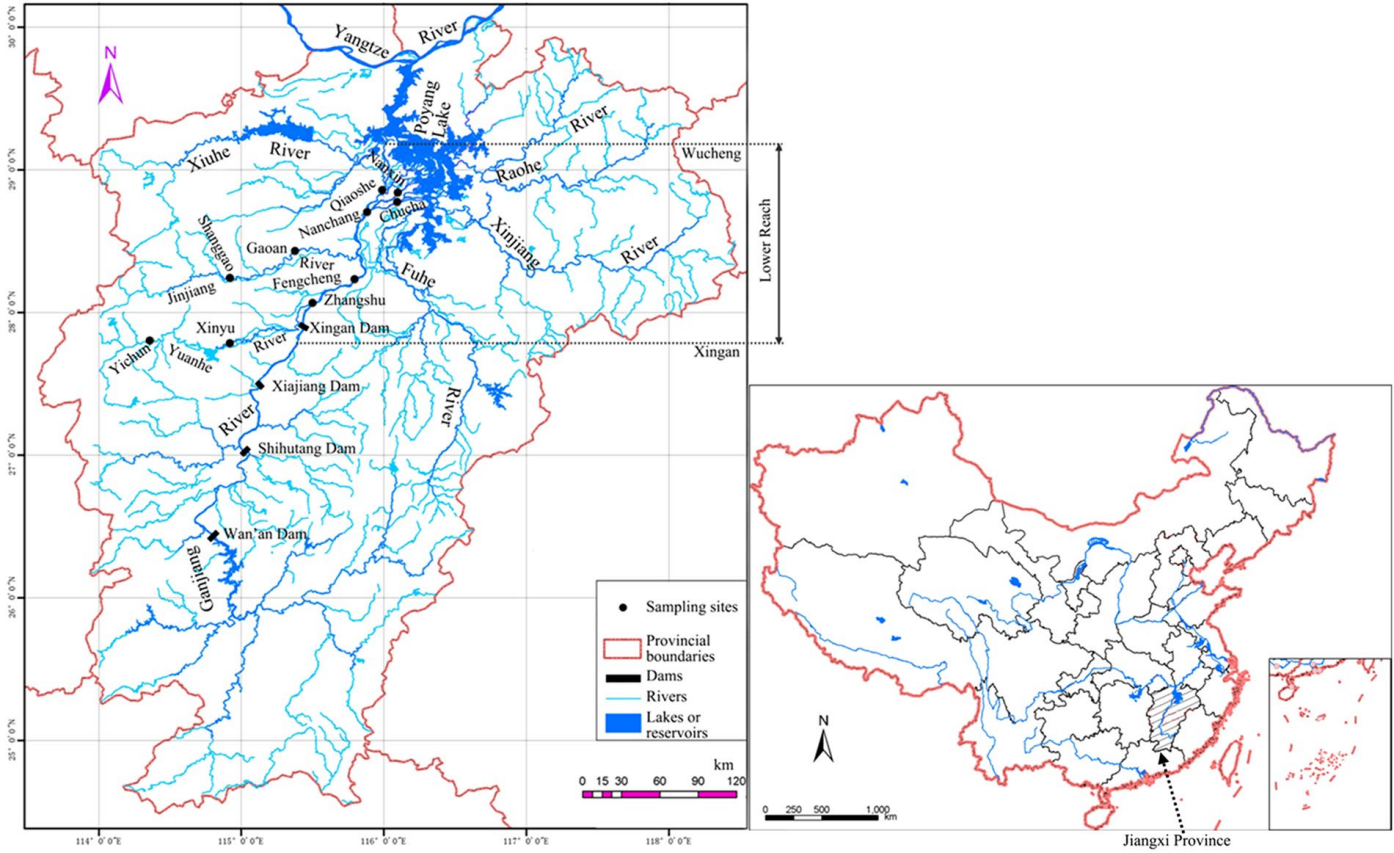
Distribution of sampling sites and dams in Ganjiang River, Jiangxi Province, China.

Since the 1990s, four water control projects (Wan’an Dam, Shihutang Dam, Xiajiang Dam and Xingan Dam) have been constructed across the Ganjiang River (Fig. 1). Habitat loss and degradation from damming and sand excavation, overfishing (e.g. electro-fishing) and other anthropogenic activities have caused a sharp decrease in fish stock in the Ganjiang River. Although a number of studies have examined the Ganjiang River basin, most focus on the middle reaches of the river^6,14–20^, with few studies aimed at fish species in the lower reaches.

The aim of this study was to: (1) describe the species composition and distribution of fish fauna in the lower reaches of the Ganjiang River, (2) analyse the spatial-temporal variation in fish assemblages, and (3) determine the changes in environmental factors that affect these assemblages. The results will help form the basis for sustainable exploitation and protection of freshwater fish in the lower reaches of the Ganjiang River.

## Results

### Species composition

Eighty-four species in total were collected from the 10 sampling sites (12,680 individuals weighing 354.09 kg), and categorized into seven orders comprising 15 families (Table 1). Of these, the most species-rich order was Cypriniformes (two families, 58 species), followed by Perciformes (five families, 10 species), Siluriformes (three families, 10 species) and Synbranchiformes (two families, three species). Meanwhile, Clupeiformes, Osmeriformes and Beloniformes were represented by only one family and one species each (Table 1).

**Table 1:** The species composition and distribution of fish in the lower reaches of Ganjiang River, showing the total number of individuals (*N*), total biomass (*W*), and index of relative importance (*IRI*).

Order/Family/Species	Sampling site										*N*	*W* (g)	*IRI* (%)
	YC	XY	SG	GA	ZS	FC	NC	QS	NX	CC			
**CLUPEIFORMES**													
**Engraulidae**													
*Coilia ectenes* Jordan *et* Seale, 1905						+	+	+	+	+	230	2465	0.8857
**CYPRINIFORMES**													
**Cyprinidae**													
*Opsariichthys bidens* Günther, 1873	+			+	+						12	527	0.0304
*Zacco platypus* (Temminck *et* Schlegel, 1846)					+					+	9	217	0.0066
*Ctenopharyngodon idella* (Valenciennes, 1844)	+			+	+				+	+	22	27693	1.6358
*Squaliobarbus curriculus* (Richardson, 1846)	+	+	+	+	+		+	+	+		26	5815	0.4576
*Chanodichthys dabryi dabryi* (Bleeker, 1871)		+					+				9	417	0.0094
*Chanodichthys mongolicus mongolicus* (Basilewsky, 1855)			+	+			+	+		+	8	900	0.0473
*Chanodichthys oxycephalus* Bleeker, 1871*						+			+		4	797	0.0191
*Culter alburnus* Basilewsky, 1855			+	+	+		+	+	+	+	99	8375	1.0952
*Cultrichthys erythropterus* (Basilewsky, 1855)			+							+	7	228	0.006
*Hemiculter bleekeri* Warpachowsky, 1887*			+	+		+	+	+	+		134	1548	0.3388
*Hemiculter leucisculus* (Basilewsky, 1855)		+		+	+	+	+	+	+	+	1606	12222	8.5462
*Hemiculterella wui* (Wang, 1935)*			+				+				14	185	0.0082
*Megalobrama amblycephala* Yih, 1955*									+		2	2584	0.0368
*Megalobrama terminalis* (Richardson, 1864)					+		+	+	+		5	2066	0.077
*Megalobrama skolkovii* Dybowski, 1872										+	2	160	0.0015
*Parabramis pekinensis* (Basilewsky, 1855)				+	+	+	+	+	+	+	174	20402	2.8339
*Pseudohemiculter dispar* (Peters, 1880)			+						+		17	612	0.0153
*Pseudolaubuca sinensis* Bleeker, 1864				+	+		+	+		+	53	611	0.0744
*Sinibrama macrops* (Günther, 1868)*	+		+				+		+	+	29	752	0.0663
*Pseudobrama simoni* (Bleeker, 1864)*		+					+	+	+	+	2844	29027	10.8102
*Xenocypris argentea* Günther, 1868		+	+	+	+	+	+	+	+	+	740	33848	10.1983
*Xenocypris davidi* Bleeker, 1871					+		+				6	547	0.01
*Aristichthys nobilis* (Richardson, 1845)		+		+	+					+	8	9647	0.3443
*Hypophthalmichthys molitrix* (Valenciennes, 1844)		+		+	+			+		+	26	20075	1.1598
*Acheilognathus barbatulus* Günther, 1873			+					+	+		42	217	0.0397
*Acheilognathus barbatus* Nichols, 1926*				+							3	28	0.0008
*Acheilognathus chankaensis* (Dybowsky, 1872)	+	+	+	+							145	757	0.3433
*Acheilognathus hypselonotus* (Bleeker, 1871)*								+			2	15	0.0005
*Acheilognathus imberbis* Günther, 1868*	+	+									45	60	0.0189
*Acheilognathus macropterus* (Bleeker, 1871)				+			+	+	+	+	414	2048	1.2639
*Acheilognathus meridianus* (Wu, 1939)*				+							1	9	0.0003
*Acheilognathus polylepis* (Woo, 1964)*				+							9	64	0.0022
*Acheilognathus tonkinensis* (Vaillant, 1892)				+							9	137	0.0028
*Rhodeus ocellatus* (Kner, 1867)*	+						+				29	37	0.0121
*Rhodeus sinensis* (Günther, 1868)*	+							+			489	479	0.4052
*Abbottina rivularis* (Basilewsky, 1855)	+	+							+	+	121	1162	0.1941
*Hemibarbus labeo* (Pallas, 1776)			+	+			+				20	2305	0.0602
*Hemibarbus maculatus* Bleeker, 1871	+		+	+	+	+		+			43	1721	0.268
*Microphysogobio microstomus* Yue, 1995*	+	+					+	+		+	30	118	0.0342
*Microphysogobio kiatingensis* (Wu, 1930)*				+							4	33	0.001
*Pseudogobio guilinensis* Yao *et* Yang, 1982*				+							4	115	0.0016
*Pseudogobio vaillanti* (Sauvage, 1878)*			+								7	156	0.0025
*Pseudorasbora parva* (Temminck *et* Schlegel, 1846)	+										28	67	0.0182
*Rhinogobio typus* Bleeker, 1871*					+	+					21	4626	0.0892
*Sarcocheilichthys kiangsiensis* Nichols, 1930*	+	+		+	+		+	+	+	+	44	501	0.1846
*Sarcocheilichthys nigripinnis* (Günther, 1873)		+	+	+	+		+	+	+	+	89	1173	0.3901
*Sarcocheilichthys parvus* Nichols, 1930*			+								1	8	0.0003
*Sarcocheilichthys sinensis sinensis* Bleeker, 1871*				+	+	+	+	+	+	+	32	621	0.1289
*Saurogobio dabryi dabryi* Bleeker, 1871		+	+	+	+	+	+	+	+	+	843	10413	6.2788
*Squalidus argentatus* (Sauvage *et* Dabry, 1874)	+	+	+	+	+	+	+	+			873	6024	4.338
*Gobiobotia filifer* (Garman, 1912)*				+	+	+					113	434	0.077
*Cyprinus carpio* Linnaeus, 1758	+	+		+	+	+	+	+	+	+	493	73125	17.194
*Carassius auratus auratus* (Linnaeus, 1758)	+	+	+	+		+	+	+	+	+	439	16118	4.6077
*Spinibarbus hollandi* Oshima, 1919				+							3	704	0.011
**Cobitidae**													
*Cobitis macrostigma* Dabry de Thiersant, 1872*									+	+	2	12	0.001
*Cobitis sinensis* Sauvage *et* Dabry de Thiersant, 1874			+							+	23	45	0.0098
*Misgurnus anguillicaudatus* (Cantor, 1842)	+	+					+			+	146	1701	0.3289
*Parabotia banarescui* (Nalbant, 1965)*				+	+	+			+		53	610	0.1041
**SILURIFORMES**													
**Amblycipitidae**													
*Liobagrus anguillicauda* Nichols, 1926*	+										2	15	0.001
**Siluridae**													
*Silurus asotus* Linnaeus, 1758		+	+	+	+	+	+	+	+	+	73	24905	2.8242
**Bagridae**													
*Hemibagrus macropterus* (Bleeker, 1870)*			+	+	+	+					11	213	0.0184
*Leiocassis crassilabris* Günther, 1864*			+	+	+	+					18	608	0.0314
*Pelteobagrus fulvidraco* (Richardson, 1846)	+		+	+	+		+	+	+	+	182	2432	0.9924
*Pelteobagrus nitidus* (Sauvage *et* Dabry, 1874)*			+	+	+	+	+	+	+	+	689	5152	2.4628
*Pelteobagrus vachellii* (Richardson, 1864)				+		+	+		+	+	30	1359	0.1394
*Pseudobagrus albomarginatus* (Rendahl, 1928)*							+				1	6	0.0002
*Pseudobagrus tenuis* (Günther, 1873)*				+			+	+	+		11	79	0.0138
*Pseudobagrus ussuriensis* (Dybowski, 1872)						+					1	32	0.0004
**OSMERIFORMES**													
**Salangidae**													
*Hemisalanx brachyrostralis* (Fang, 1934)*								+			1	12	0.0003
**BELONIFORMES**													
**Hemiramphidae**													
*Hyporhamphus intermedius* (Cantor, 1842)					+	+	+	+		+	67	167	0.0729
**SYNBRANCHIFORMES**													
**Synbranchidae**													
*Monopterus albus* (Zuiew, 1793)							+				2	94	0.0021
**Mastacembelidae**													
*Mastacembelus armatus* (Lacépède, 1800)	+										11	89	0.0028
*Sinobdella sinensis* (Bleeker, 1870)	+						+			+	43	552	0.0374
**PERCIFORMES**													
**Percichthyidae**													
*Siniperca chuatsi* (Basilewsky, 1855)			+	+	+	+	+	+	+		32	6285	0.6028
*Siniperca kneri* Garman, 1912	+			+	+	+	+	+	+		347	2081	0.7217
*Siniperca obscura* Nichols, 1930*						+		+			4	314	0.009
*Siniperca roulei* Wu, 1930*						+					7	215	0.0029
**Odontobutidae**													
*Odontobutis sinensis* Wu, Chen *et* Chong, 2002*	+	+					+			+	73	500	0.1811
*Micropercops swinhonis* (Günther, 1873)*	+										133	139	0.0828
**Gobiidae**													
*Rhinogobius giurinus* (Rutter, 1897)	+	+		+		+	+			+	223	651	0.4213
**Osphronemidae**													
*Macropodus chinensis* (Bloch, 1790)*	+										1	1	0.0002
*Macropodus opercularis* (Linnaeus, 1758)	+										8	23	0.0035
**Channidae**													
*Channa argus* (Cantor, 1842)				+			+			+	4	804	0.0192

^*^Endemic to China^4,5^.

The dominant family was Cyprinidae (54 species, 64.29% of the total species collected), followed by Bagridae (eight species, 9.52%), Cobitidae and Percichthyidae (four species and 4.76%, respectively), Mastacembelidae, Odontobutidae and Osphronemidae (two species and 2.38% each). Engraulidae, Amblycipitidae, Siluridae, Salangidae, Hemiramphidae, Synbranchidae, Gobiidae and Channidae were all represented by one species each (Table 1).

A total of 36 endemic species (42.86% of the total species collected) in eight families were identified. Cyprinidae was the dominant family (22 species), followed by Bagridae (five species), Cobitidae, Percichthyidae and Odontobutidae (two species each). Meanwhile, Amblycipitidae, Salangidae and Osphronemidea were each represented by one species (Table 1).

The dominant species were Cyprinus carpio (IRI, 17.19%), Pseudobrama simoni (IRI, 10.81%) and Xenocypris argentea (IRI, 10.20%); the common species were Hemiculter leucisculus (IRI, 8.55%), Saurogobio dabryi dabryi (IRI, 6.28%), Carassius auratus auratus (IRI, 4.61%), Squalidus argentatus (IRI, 4.34%), Parabramis pekinensis (IRI, 2.83%), Silurus asotus (IRI, 2.82%), Pelteobagrus nitidus (IRI, 2.46%), Ctenopharyngodon idella (IRI, 1.64%), Acheilognathus macropterus (IRI, 1.26%), Hypophthalmichthys molitrix (IRI, 1.16%) and Culter alburnus (IRI, 1.10%). The abundance and biomass of these 14 species accounted for 73.62% of the total individuals and 81.74% of the total biomass (Table 1).

### Species distribution

The general distribution of species collected in the 10 sampling sites is shown in Table 2. Twenty species appeared in more than 60% of sites, while 37 species were recorded in only one or two sites.

**Table 2:** Temporal variations in fish species, individuals, biomass and diversity indexes in the lower reaches of the Ganjiang River.

Season	Total number of species	Total number of individuals	Total biomass (g)	Margalef species richness index	Shannon−Wiener diversity index	Pielou evenness index
Spring	56	2982	65307	6.87	2.93	0.70
Summer	59	3735	124712	7.05	3.22	0.69
Autumn	57	3581	109822	6.84	2.85	0.75
Winter	49	2382	54250	6.17	2.80	0.80

**Figure 2:**
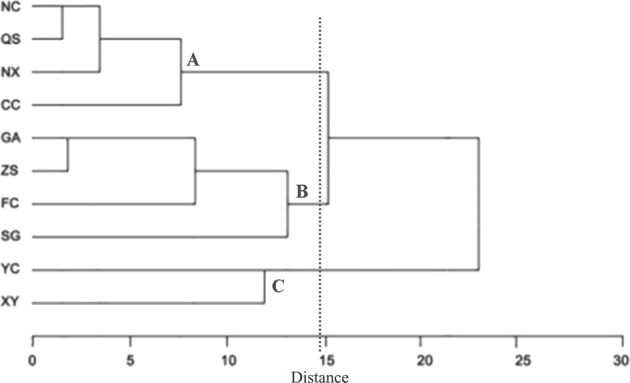
Cluster analyses of the fish species collected at 10 sampling sites in the lower reaches of the Ganjiang River.

Cluster analysis divided the fish species into three significantly different groups (Fig. 2). Group A comprised sites NC, QS, NX and CC, all of which were located in the estuary of Ganjiang River. Seven species (*Megalobrama amblycephala*, *Megalobrama skolkovii*, *Acheilognathus hypselonotus*, *Cobitis macrostigma*, *Pseudobagrus albomarginatus*, *Hemisalanx brachyrostralis* and *Monopterus albus*) were recorded in group A only. Group B covered sites GA, ZS, FC and SG, all of which were located in Jinjiang River and lower mainstream regions of Ganjiang River. Fifteen species (*Acheilognathus barbatus*, *Acheilognathus meridianus*, *Acheilognathus polylepis*, *Acheilognathus tonkinensis*, *Microphysogobio kiatingensis*, *Pseudogobio guilinensis*, *Pseudogobio vaillanti*, *Rhinogobio typus*, *Sarcocheilichthys parvus*, *Gobiobotia filifer*, *Spinibarbus hollandi*, *Hemibagrus macropterus*, *Leiocassis crassilabris*, *Pseudobagrus ussuriensis* and *Siniperca roulei*) were found in these sites only. Group C comprised sites XY and YC, which were located in Yuanhe River. Seven species (*Acheilognathus imberbis*, *Pseudorasbora parva*, *Liobagrus anguillicauda*, *Mastacembelus armatus*, *Micropercops swinhonis*, *Macropodus chinensis* and *Macropodus opercularis*) appeared in this group only.

### Spatial-temporal variation in the fish assemblage

The highest fish species richness, species abundance and species biomass as well as Margalef species richness index and Shannon−Wiener diversity index occurred in the summer, while lowest values observed in the winter. In contrast, the highest Pielou evenness index was observed in the winter, with lowest values in the summer (Table 2).

Highest species richness was observed in sites GA and NC (41 fish species, respectively), while lowest values (20 species) were observed in site XY. Highest abundance (2840 individuals) was observed in site NC, while lowest (237 individuals) was observed in site SG. Highest biomass (63058 g) was observed in site ZS, while lowest (3762 g) was observed in site YC. The species diversity indices also differed among sampling sites. The highest Margalef species richness index (6.90) was observed in site GA, while the lowest (3.20) was observed in site FC. The highest Shannon−Wiener diversity index (2.93) and Pielou evenness index (0.78) were observed in site GA, while the lowest values were observed in site NX (1.54 and 0.44, respectively) (Table 3).

**Table 3:** Spatial variations in fish species, individuals, biomass and diversity indexes in the lower reaches of the Ganjiang River.

Site	Total number of species	Total number of individuals	Total biomass (g)	Margalef species richness index	Shannon−Wiener diversity index	Pielou evenness index
YC	28	1173	3762	3.68	2.22	0.67
XY	20	479	34313	3.24	1.98	0.65
SG	27	237	11719	4.57	2.54	0.78
GA	41	439	25024	6.90	2.93	0.78
ZS	31	690	63058	4.44	2.53	0.74
FC	27	1600	44851	3.20	1.76	0.55
NC	41	2840	57931	5.03	2.33	0.63
QS	34	1720	39212	4.43	2.11	0.60
NX	32	2070	55441	4.06	1.54	0.44
CC	36	1432	18780	4.82	2.57	0.72

**Figure 3:**
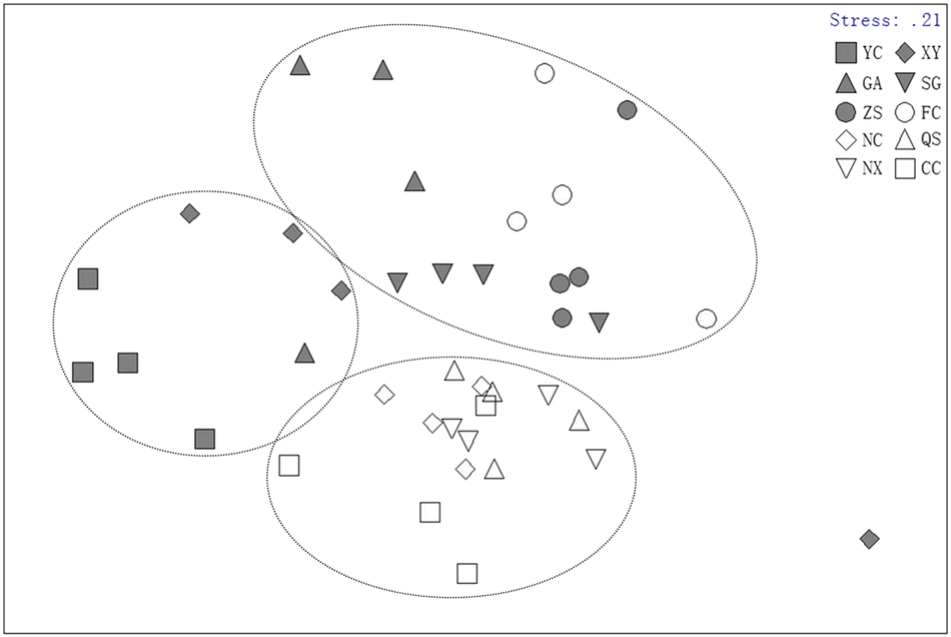
Non-metric multidimensional scaling (NMDS) of the sampling sites based on abundance data.

Non-metric multidimensional scaling ordination also clearly separated the samples into three groups by sampling sites (Fig. 3). Meanwhile, one-way ANOSIM also revealed a highly significant effect of sampling site (Global test *R* = 0.448, *P* = 0.001) based on fish abundance data.

### Characteristics of the ABC

**Figure 4:**
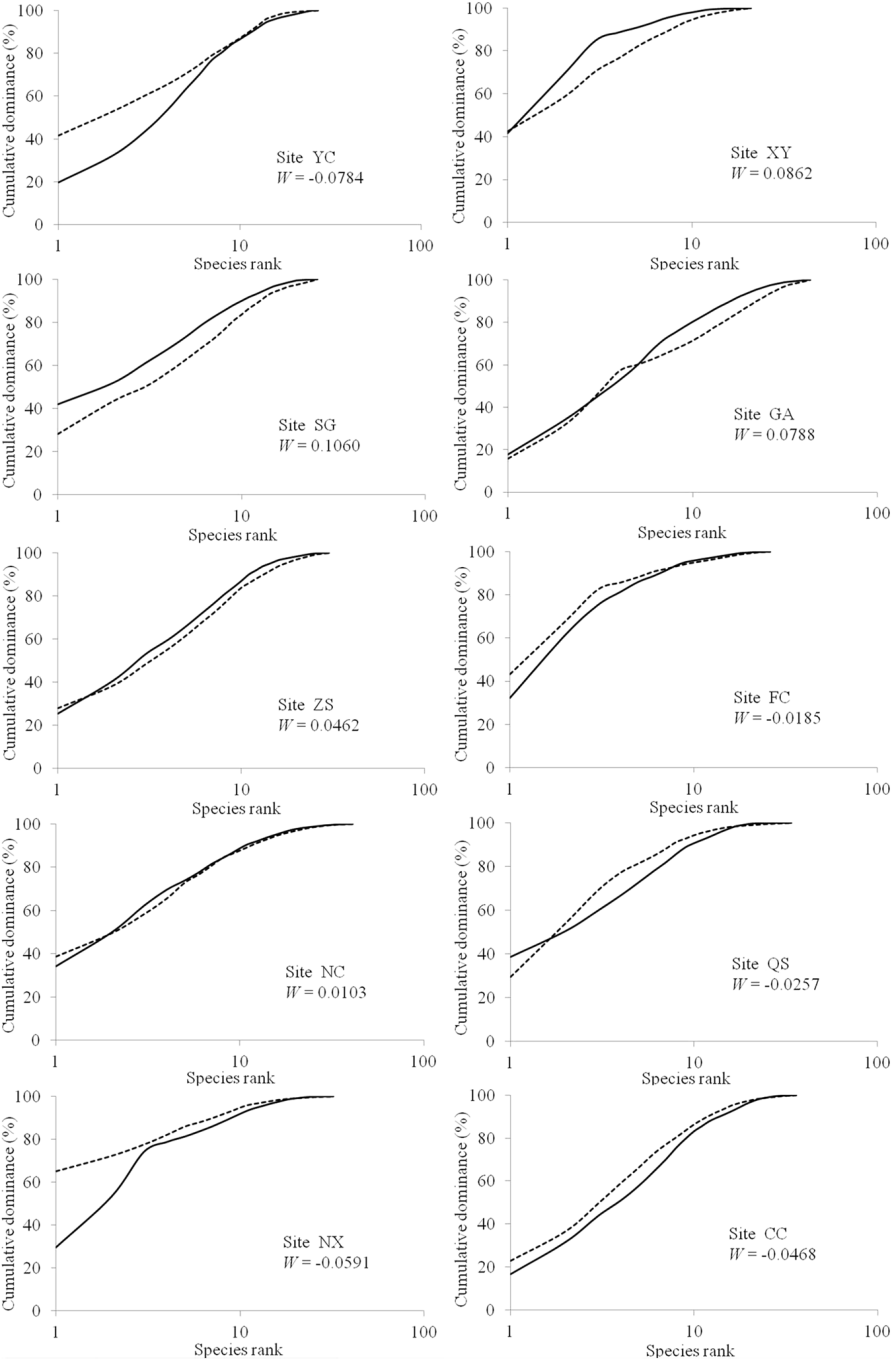
Comparison of abundance-biomass curves for the 10 sampling sites. Dashed line: abundance cumulative frequency; Solid line: biomass cumulative frequency. W-Statistics represent the closeness between abundance and biomass curves.

Abundance curves of fish communities at sites YC, FC, QS, NX and CC lay either above the biomass curve, or the two curves crossed, and the W-statistics were negative. For the remaining five sampling sites, the biomass curves lay above the abundance curves in some places and had small positive W values (Fig. 4). These findings suggest moderate disturbance.

### Correlation between environmental factors and fish composition

Water temperature (WT) and the dissolved oxygen (DO) content differed by seasons; however, no obvious spatial or temporal differences in pH were observed. Maximum WT and DO values were 32.7 °C (site NX in summer) and 12.7 mg/L (site CC in autumn), while lowest values were 8.8 °C (site FC in winter) and 3.9 mg/L (site GA in summer). In contrast, the mean WT and DO did not differ significantly within sampling sites. The site average WT ranged between 19.7 °C and 22.1 °C (mean ± SD, 20.8 ± 0.7 °C), while the mean DO (±SD) was 7.2 ± 0.5 mg/L, and ranged between 6.3 and 8.0 mg/L. The maximum and minimum pH were 8.1 and 6.8, respectively, and the site average pH ranged from 7.0 to 7.8 (mean ± SD, 7.5 ± 0.3) (Table 4).

**Table 4:** Physic-chemical parameters of the survey sites.

Site	Water temperature (°C)		Dissolved oxygen (mg/L)		pH	
	Range	Mean ± SD	Range	Mean ± SD	Range	Mean ± SD
YC	10.3–30.6	20.1 ± 7.4	5.8–10.3	8.0 ± 4.3	7.6–7.8	7.8 ± 0.2
XY	11.9–30.4	20.6 ± 6.7	5.4–9.1	7.3 ± 1.8	7.4–8.1	7.6 ± 0.2
SG	10.7–31.8	21.6 ± 8.1	4.8–9.5	6.9 ± 1.9	7.4–7.9	7.6 ± 1.9
GA	10.2–30.5	20.8 ± 7.4	3.9–9.9	6.3 ± 2.2	7.4–8.0	7.6 ± 0.2
ZS	10.3–31.3	21.1 ± 7.5	5.2–10.3	7.2 ± 2.1	7.6–7.8	7.7 ± 0.1
FC	8.8–29.7	19.7 ± 7.5	4.5–8.7	7.1 ± 1.7	7.4–7.9	7.6 ± 0.2
NC	9.4–31.4	20.9 ± 8.5	5.6–11.7	7.8 ± 2.3	6.8–7.9	7.4 ± 0.4
QS	9.8–27.3	22.1 ± 9.4	6.2–9.6	7.1 ± 1.4	6.8–7.1	7.0 ± 0.1
NX	9.2–32.7	20.9 ± 8.9	6.1–7.3	6.9 ± 0.5	6.8–7.2	7.1 ± 0.2
CC	9.6–30.5	20.6 ± 8.0	5.5–12.7	7.7 ± 3.0	7.2–7.5	7.4 ± 0.1

**Figure 5:**
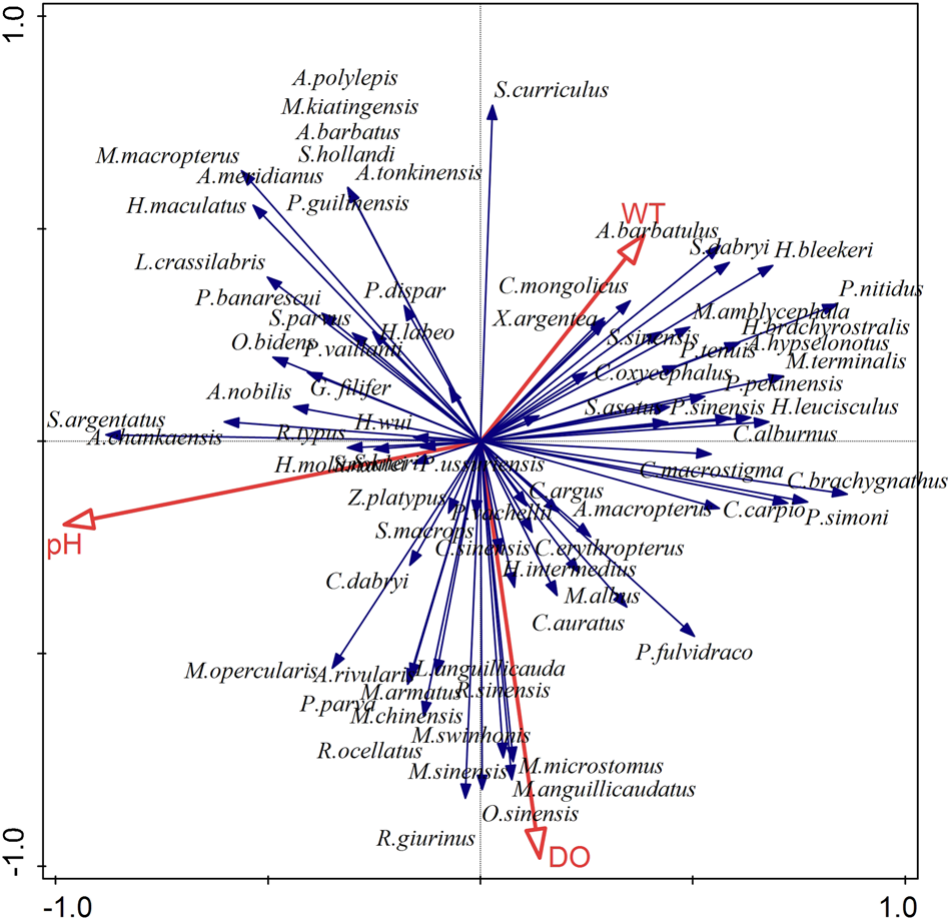
Ordination bi-plot of the fish species assemblages and environmental variables obtained by RDA across sampling periods and sites.

RDA was subsequently used to generate bi-plots after extracting and integrating the data from the fish community indices with the environmental data (WT, DO and pH) (Fig. 5). The first axis of the cumulative percentage of variance of the species–environmental relationship was 26.85%, with four axes accounting for 67.34%. Overall, these findings suggest that WT, DO and pH had a significantly effect on fish distribution and assemblage composition in the study area (P < 0.05).

## Discussion

### General characteristics of the fish fauna and assemblage

Eighty-four fish species belonging to seven orders and 15 families were collected from the lower reaches of the Ganjiang River, the majority belonging to Cypriniformes and Cyprinidae. The most species-rich order and family also represented the richest order and family in the Yangtze River Basin and China^2,4^. Overall, 36 species were endemic to China. The total number of species and endemic species represented 38 and 27% of the total for Jiangxi Province^5^, and 20 and 11% of these in the Yangtze River Basin, respectively^2,4^. Fish biodiversity in the study area is therefore also important in the larger context of Jiangxi Province and the Yangtze River Basin.

Further analyses revealed that most species were omnivorous, pelagic or settled fish that lay pelagic eggs. However, the Margalef species richness index and Shannon−Wiener diversity index were low compared with adjacent watershed areas, such as middle reaches of Ganjiang River^15,17,20^ and Poyang Lake^21–24^.

The number of species with an average body weight of less than 200 g per individual represented approximately 90% of the total species richness. Meanwhile, smaller individuals (<200 g) represented 98.5% of the total abundance. Moreover, ABC analyses suggested moderate disturbance of fish population in this area. The findings also revealed a small proportion of large commercial fish species and larger numbers of small species in the sample catches, indicating an obvious low age trend. Taken together, these findings further suggest that fish resources in the lower reaches of the Ganjiang River are reaching exhaustion.

### Current threats

Disturbances resulting from dam construction, sand excavation and overfishing are the most significant threats to fish biodiversity in this area. For example, construction of Wan’an dam has affected connectivity in the river, altering the overall hydrology, and disrupting migration of fish species from connecting lakes as well as the velocity and floating distance of drifting eggs. A decline in *M. reevesii* yield has also been observed in the Ganjiang River^6^, due to reduced water inflow, which in turn is destroying breeding habitats^25^. Historically, 12 spawning grounds of four domestic fish species once existed in the middle reaches of Ganjiang River^6^. However, construction of Wan’an, Shihutang, Xiajiang and Xingan dams has caused flooding of many of these spawning areas^7^.

Meanwhile, overfishing is threatening traditional fisheries, with pressure to adopt modern harvesting activities. This process is occurring throughout all major river systems in China, especially the Yangtze River Basin^2^. Overfishing of spawning fish is the main cause of the decline in fish stock^26^, leading to the so-called of ‘fishing down the food web’ phenomenon, whereby larger elements of a multispecies fish assemblage are successively removed and replaced by smaller elements, which typically represent lower trophic levels^27^. Illegal fishing, the effects of which are relatively small-scale but wide-ranging, is also inflicting considerable damage on freshwater fisheries. For example, electrofishing has increased throughout China, providing short-term profits and an efficient collecting method^28^. Although this practice is illegal, it remains rampant, having a huge detrimental effect on fish populations across China. In the Ganjiang River, a large number of fishing methods are employed, including traps, gill nets and; electrofishing, all of which are leading to overfishing and a dramatic decrease in fish biodiversity^29,30^.

Sand extraction is also having damaging effects on fish feeding, migration and reproduction grounds^29,31,32^. For example, a 50-flod increase in water turbidity occurred from 1998 to 2004 as a result of sand excavation, causing a 0.3 km^2^ grass island to slide into Poyang Lake in 2004^33^. The combined effects of high temperatures and precipitation, mountainous and hilly terrain, and large-scale construction has also resulted in severe soil erosion and water losses in Jiangxi Province^34,35^. As a resulted, widespread alterations and loss of habitat have occurred, with subsequent decreases in fish resources^31,36–39^.

### Conservation recommendations

Although a variety of measures aimed at conserving fish biodiversity in the Ganjiang River have been implemented at a local and national level, these efforts remain inadequate and further conservation strategies are essential. Protection of freshwater fish should be carried out based on a comprehensive understanding of large-scale species richness patterns as well as patterns of endemism^40^. This approach would provide a platform for evaluating the current status of freshwater fish resources. This updated status information is essential in determining appropriate strategies of conservation management. Such data would also help highlight the overall status of freshwater fish in Jiangxi Province. Government-sponsored, national-scale screening of freshwater fish species in Jiangxi Province is therefore recommended. Furthermore, a system that responds to real-time threats such as dam construction would also help the implementation of conservation strategies prior to disruption.

The development of protected areas is also essential in preventing habitat loss and degradation; however, few such areas have been created for freshwater habitats. Instead, freshwater habitats tend to be protected incidentally due to inclusion within terrestrial reserves. Saunders *et al*.^41^ suggested that freshwater species and habitats be directly conserved through the creation of freshwater protected areas. Recently, such areas have been established globally, playing an important role in conserving freshwater fish diversity^42–44^. In Jiangxi Province, approximately 156 protected areas have been established for the conservation of plants, animals and wetlands; however, no freshwater areas or fish passage facilities in Ganjiang River have yet to be included^5^. Protection of fish biodiversity therefore also requires the immediate establishment of freshwater protected areas.

According to Chinese Fishery Laws implemented in 1987, fishing is annually prohibited for two months from June 1st to July 31st along the Xiajiang, Xingan, Jishui and Ji’an reaches of the Ganjiang River. This initiative has played an important role in restoring populations of *M. reevesii* in the Yangtze River^6^. Fishing is also forbidden during the breeding season in mainstream rivers and tributaries in Jiangxi Province. However, this law is not widely respected, and many local residents fish throughout the year using traps and electro-fishing techniques^5^. To more effectively protect fish diversity and resources, enforcement of these laws is therefore required.

In addition to the above, the following conservation measures are also recommended: (1) restocking of economically important fish species; (2) enforced implementation of a close season; and (3) active development of sustainable aquaculture^4,5,45,46^.

## Methods

### Ethics statement

The study was approved by the Institutional Animal Care and Use Committee (IACUC) of Nanchang University, Jiangxi, China. All necessary permits were obtained for the described field studies from the IACUC of Nanchang University and the Yangtze River Fishery Administration of China. The handling of fish was also conducted in accordance with the guidelines on the care and use of animals for scientific purposes set by IACUC of Nanchang University, Jiangxi, China. All methods were carried out in accordance with relevant guidelines and regulations.

### Study area

Jiangxi Province which is located in the middle and lower reaches of the Yangtze River (24°29′14′′–30°04′41′′N to 113°34′36′′–118°28′36′′E) covers an area of approximately 166,900 km^2^. Its northern areas are relatively flat, while remaining areas are surrounded by mountains. Main rivers in Jiangxi Province are Ganjiang, Xinjiang, Fuhe, Raohe and Xiuhe, all of which flow into Poyang Lake and drain into the Yangtze River^6^.

Of these^47^, Ganjiang River is the longest, spanning more than 823 km and with a drainage basin of 82,809 km^2^. The riverhead is located in Shiliaodong (Yangdi Town: 116°22′E and 25°57′N), while its estuary is located in Wangjiangting (Wucheng Town: 116°01′E and 29°11′N). Its basin presents a mid-subtropical humid climate, with annual average precipitation of approximately 1580.8 mm and mean annual river runoff of about 2125 m^3^/s^47^.

The lower reaches of the Ganjiang River run from Xingan to the mouth of the river, covering approximately 208 km. This segment of river meanders among plains and hills, and has two tributaries (Yuanhe and Jinjiang River) draining into it. These lower reaches flow through Nanchang then divide into three branches: northern, middle and southern branches (Fig. 1). Yuanhe River, which originates to the west of the Wukong Mountain Range (114°10′E and 27°27′N), with its estuary located in Hehuguan (Zhangjiashan Town: 115°29′E and 28°04′N), flows from southwest to northeast and empties into Ganjiang River. Meanwhile, Jinjiang River originates to the east of the Mufu Mountain Range (114°01′E and 27°57′N) with its estuary located in Ruihe (Xinjian County: 115°49′E and 28°25′N), and has a similar direction of flow as the Yuanhe River^47^.

### Survey sites

Sampling site selection was based on representative habitat types and accessibility during the study period. Local knowledge and previous surveys were also referred to in determining the final sampling sites. As a result, 10 sites were established in the lower reaches of the Ganjiang River: YC: Yichun (114°21′05″E, 27°48′09″N), XY: Xinyu (114°56′03″E, 27°47′46″N), SG: Shanggao (114°57′51″E, 28°14′44″N), GA: Ganan (115°22′09″E, 28°25′07″N), ZS: Zhangshu (115°33′13″E, 28°04′38″N), FC: Fengcheng (115°46′39″E, 28°11′56″N), NC: Nanchang (115°49′25″E, 28°39′50″N), QS: Qiaoshe (115°59′20″E, 28°50′38″N), NX: Nanxin (116°4′19″E, 28°47′52″N), CC: Chucha (116°5′56″E, 28°46′12″N) (Fig. 1). Physico-chemical parameters (water temperature, the dissolved oxygen content and pH) were measured at each sampling site using a hand-held YSI multi-meter.

### Sampling method

Fish samples were obtained quarterly, in April, July, October and January, 2017 to 2018. The water depth in the study area was more than 1 m, and therefore, local fishermen were hired to catch the fish samples. Each site was sampled using a ground cage (5 m long × 0.5 m × 0.5 m, 5 mm mesh) and a gillnet (50 × 3 m) comprised of five panels (1.5, 3, 4.5, 6 and 7.5-cm bar mesh, respectively). Fishing was carried out overnight for approximately 10 h. Additional collection using a trawling net (100 m × 2 m, 5 cm mesh) was also performed at each site for about 1 h.

Where possible, fish were identified on collection then released. Those that were not were preserved in 10% formalin solution and taken to the laboratory for identification. Identification was carried out according to Zhu^48^, Chen^49^, Chu *et al*.^50^ and Yue^51^. All fish specimens were deposited in the museum specimens of the fish existed at School of Life Sciences, Nanchang University.

### Statistics analyses

Fish dominance in each catch was determined by the index of relative importance (*IRI*) based on the number percentage, weight percentage and frequency of occurrence^52^:

$$IR{I}_{i}=( \% {N}_{i}+ \% {W}_{i})\times  \% {F}_{i}$$

where %*N*_*i*_ and %*W*_*i*_ represent the percentage number and percentage weight of species *i* in the total catch, respectively, and %*F*_*i*_ is the occurrence frequency of species *i*. When *IRI*_*i*_ was greater than 10%, it suggested that the species *i* was dominant, while 1% < *IRI*_*i*_ < 10% suggested that species *i* was common.

Species diversity can be defined as the species richness in a certain area in a certain period. In this study, diversity indices were used to measure the spatial-temporal variation in fish species diversity as follows^53,54^:

$$\begin{array}{rcl}{\rm{Margalef}}\,{\rm{species}}\,{\rm{richness}}\,{\rm{index}}:D & = & (S-1)/\mathrm{ln}\,N\\ \mathrm{Shannon}-\mathrm{Wiener}\,{\rm{diversity}}\,{\rm{index}}:H^{\prime}  & = & -\sum {P}_{i}\,\mathrm{ln}\,{P}_{i}\\ {\rm{Pielou}}\,{\rm{evenness}}\,{\rm{index}}:J^{\prime}  & = & H^{\prime} /\mathrm{ln}\,S\end{array}$$

where *S* is the number of species, *N* is the sum of individual number of species in the community, *N*_*i*_ is the individual number of species *i*, and *P*_*i*_ is the ratio of *N*_*i*_ to *N*.

A dataset covering all species collected at each site was then constructed, and similarity analyses were carried out based on the presence (1) or absence (0) of each species at each site^5^. Pairwise similarities among sites were then computed in order to create a similarity coefficient matrix. The hierarchical cluster, furthest-neighbour method with squared Euclidean distance was then used for cluster analysis based on the matrix. All analyses were performed using SPSS 13.0 software.

One-way analysis of similarities (ANOSIM) was used to determine significant differences in under non-metric multidimensional scaling (NMDS) ordination. First, a global R statistic was calculated to determine significant differences between all groups (analogous to the global F test in ANOVA). Significant differences at a global level were then determined using pairwise comparisons between sample groups to test for differences between pairs. In the global test, significance was set at P < 0.05^55^. All multivariate analyses were performed using Plymouth Routines in the Multivariate Ecological Research (PRIMER v5.0) software.

Abundance-biomass curves (ABC) were also obtained to determine differences in the community disturbance level. The ABC method, which is based on r and k-selection, was first proposed by Warwick^56^ as a variant of K-dominance curves^57^ to determine the effect of disturbances to invertebrate communities^58^. Communities in a stable state are dominated by k-selected species with slow growth rates, large body sizes, late maturation, and population sizes close to the environmental carrying capacity. In contrast, opportunistic species with fast growth rates, small body sizes and highly variable population sizes (r-selected) dominate systems where disturbance is recurrent or has occurred recently. These characteristics translate into two distinct patterns: a biomass curve lying on top of the abundance curve in undisturbed environments and the opposite in disturbed environments^59^. W-Statistics vary between −1 and +1 representing the difference between abundance and biomass curves. Values close to +1 represent a higher biomass than abundance curve (i.e. a stable environment), while values close to −1 suggest a reverse pattern (i.e. a disturbed environment). Meanwhile, values close to 0 represent a moderately disturbed community^60^.

Redundancy analysis (RDA) was also carried out to analyse the correlations between fish species composition and the measured environmental factors. Species composition and environmental data were log 10(X + 1)-transformed to meet the assumptions of multivariate normality and limit the effect of extreme data. All ordinations were carried out using CANOCO 5.0^61^.
